# Medicinal Use of *Lepidium sativum* and *Nigella sativa* Seeds in North Shewa Zone, Amhara and Oromia Regions, Ethiopia

**DOI:** 10.1155/bmri/2989902

**Published:** 2026-10-12

**Authors:** Daniel Molla Melese, Wondyefraw Mekonen, Abebaye Aragaw

**Affiliations:** ^1^ Department of Biomedical Science Asrat Woldeyes Health Science Campus Debre Berhan University Debre Berhan Ethiopia dbu.edu.et; ^2^ Department of Physiology College of Health Sciences Addis Ababa University Addis Ababa Ethiopia aau.edu.et

**Keywords:** Ethiopia, frequency of citation, informant consensus factor, *Lepidium sativum*, *Nigella sativa*

## Abstract

**Background:**

*Nigella sativa* seeds have been used for the treatment of ailments. *Lepidium sativum* is another spice used in the treatment of asthma, cough, and bleeding piles. Despite their significance, a comprehensive synthesis of their ethnobotanical uses in Ethiopia remains limited. Therefore, this study was designed to carry out an ethnobotanical investigation on *L. sativum* and *N. sativa* seeds.

**Methods:**

The study was conducted in Debre Berhan, Chacha, and Sheno towns located in the North Shewa Zone of the Amhara Regional State, Ethiopia. Semistructured interviews were employed among 65 informants to obtain ethnobotanical data. Data were entered into EpiData Version 3.1, then exported to SPSS Version 26.0 for statistical analysis. Descriptive statistics, including frequencies and percentages, were used to summarize the data. Ethnobotanical indices, including informant consensus factor (ICF) and fidelity level (FL), were calculated using the standard formulas. Associations between categorical and patterns of medicinal seed use were assessed using the Pearson chi‐square test. Statistical significance was considered at *p* value of < 0.05.

**Results:**

A total of 65 individuals participated in the study. The majority of the respondents (93.8%) reported having used these seeds. Among those, 84.6% used both seeds in combination. ICF values ranged from 0.000 to 0.933 across six disease categories. The highest was recorded for digestive problems (ICF = 0.967) and general health (ICF = 0.958), with 31 and 25 use‐reports, respectively. The ethnobotanical indices showed a significant preference for the combined use of *L. sativum* and *N. sativa* with a frequency of citation (FC) value of 55 and a use value (UV) of 0.846.

**Conclusions:**

In this study, people use *L. sativum* and *N. sativa* seeds mostly for digestive problems and general health conditions. Future studies should investigate the phytochemical composition, mechanisms of action, toxicological effects, and clinical relevance of commonly used preparations of *L. sativum* and *N. sativa.*

## 1. Introduction

*Nigella sativa* belongs to the Ranunculaceae family and is an annual herb of 8–12 in. in height, with pinnate and segmented leaves [1]. Its flowers are solitary, self‐pollinating, and colored in blue and white. The capsule fruits contain numerous trigonal seeds [1]. *N. sativa* (black cumin) seeds are curved and dark brownish in color with distinct pods [2]. The estimated early world production is 600,000 t, with 86% of the share in India, followed by Iran (4%), Syria (3%), and Turkey (2%). The remaining 5% comes from different countries such as Egypt, Pakistan, and Afghanistan [3]. In Ethiopia, the annual production of black cumin was 14,730 Mt in the 2021/2022 cropping season [4].

Traditionally, black cumin seeds have been used for the treatment of ailments such as nasal congestion, headache, asthma, diabetes, toothache, arthritis, conjunctivitis, abscesses, and intestinal worms [5]. These seeds have an acerbic and bitter taste [6]. *N. sativa* (black cumin) is readily available throughout the year in the local markets [7]. Its seeds are also available in ground powder form [8]. *N. sativa* powdered fruit is used to treat bronchial asthma. Fried seeds mixed with melted butter and sniffed relieve common cold and headaches [9]. This species also acts as a spasmolytic, antimicrobial, diuretic and has cancer‐preventive properties [10].

Garden cress (*Lepidium sativum L*.) is a fast‐growing, perennial edible plant belongs to the Brassicaceae family [11]. The inflorescence is an elongating raceme. The flowers have four sepals and four white to reddish petals, with six stamens. The fruit is a broadly ovate silicula, approximately 5–6 mm long and 3–5 mm wide, and the seeds are ovoid, about 2–3 mm long [12].

*L. sativum* is also another spice used in the treatment of asthma, cough, and bleeding piles. The seeds are chewed to treat sore throat, cough, asthma, and headache, and in large quantities to induce abortion. They are also applied externally as an insect repellent. Leaves are mildly stimulant and diuretic, and useful in scorbutic diseases and liver complaints. The roots are used against syphilis. In Europe, the herb is used to treat cough and constipation, as a diuretic, and to improve the immune system [13].

*L. sativum* has been widely used to treat some ailments. The bruised seeds, mixed with lime juice, are used as a local application for the relief of inflammatory and rheumatic pains [14]. The seeds and leaves are used by local communities to treat skin conditions, colds, evil spirits, malaria, fever, wounds, tonsillitis, gingivitis, epiglottitis, stomach upsets, and swollen glands [9]. Seeds are often ground with ginger, black pepper, and black cumin, then mixed with honey and taken before breakfast to treat gastritis and as an aphrodisiac [15].

Overdose of *N. sativa* seeds might slow the process of blood clotting [16]. Thus, it makes bleeding worse. However, the exact mechanism is still under investigation. In addition to that, it might cause hypoglycemia if taken in an inappropriate amount [17]. It is also not recommended to take *N. sativa* seeds before or after surgery [18]. Despite their significance, a comprehensive synthesis of their ethnobotanical uses in Ethiopia remains limited. Therefore, this study was designed to carry out an ethnobotanical investigation on *L. sativum* and *N. sativa* seeds.

## 2. Materials and Methods

### 2.1. Study Area

The study was conducted in Debre Berhan, Chacha, and Sheno towns located in the North Shewa Zone of the Amhara and Oromia Region, Ethiopia. Debre Berhan is the administrative center of North Shewa Zone and lies approximately 130 km northeast of Addis Ababa. The town is situated at a high altitude and is known for its cool climate. It serves as a hub for education, health services, and trade for the surrounding rural communities. Chacha is a small town located along the main road between Addis Ababa and Debre Berhan. It functions as a commercial and transit point for nearby rural kebeles, with residents primarily engaged in farming, small‐scale trade, and traditional practices. Sheno is another growing town in North Shewa Zone, Oromia Region situated along the same highway. The town provides basic services, including health centers, schools, and local markets, and the population largely depends on agriculture and trade. These towns were selected as study areas due to their accessibility, population diversity, and the presence of communities that actively practice traditional medicine, making them suitable for assessing ethnobotanical knowledge and practices related to the medicinal use of *L. sativum* and *N. sativa* seeds.

### 2.2. Study Design, Study Period, and Data Collection Tools

A field survey study was employed from December 2025 to March 2026 to systematically collect information on the indigenous use of plant seeds for medicinal purposes in the study area. Before data collection, discussions were held with the informants, facilitated by governmental authorities, to explain the objectives of the study and to build trust, emphasizing the importance of the study. The informants included traditional healers (herbalists), herbal vendors, and community members who use *L. sativum* and *N. sativa* seeds. The interview was based on a checklist of questions prepared beforehand in English and translated to local languages (Amharic and Afaan Oromo). Information regarding indigenous use of the local community on traditional plant seeds was recorded. During the survey, both qualitative and quantitative data were collected to provide a comprehensive understanding of the subject. The qualitative data included sources of information, cultural practices, preparation methods, modes of administration, and perceived effectiveness of medicinal plant seeds. This information was obtained by semistructured questions and key informant interviews with traditional healers, herbal sellers, elders, and knowledgeable community members. On the other hand, quantitative data were collected to support the qualitative findings. This included measurable information such as the number of plant seed species used, frequency of use, proportions of respondents using specific medicinal seeds, and distribution patterns across individuals. Such data were collected using structured questionnaires and field inventories. By integrating both qualitative and quantitative approaches, the study ensured a holistic analysis of indigenous knowledge and practical experience of the community on the seeds.

### 2.3. Informant Selection

A total of 65 informants were selected. From these, five were key informants (one informant per kebele), which were selected purposively, and 60 (12 per kebele) of them were general informants, which were selected randomly (simple random sampling technique following lottery method). Age range of informants selected for the study were > 18 who lived 5 years and above in the study area.

### 2.4. Sample Size Determination and Sampling Techniques

Out of the 17 kebeles in the district, five were selected using purposive sampling based on their relevance to the study objectives. The sampled kebeles are Debre Berhan (Kebeles 07, 08, and 10), Chacha 01, and Sheno 01. A total of 65 (11 per kebele) respondents were selected. To ensure the quality of the ethnobotanical data, a purposive sampling technique was used. This allowed for the deliberate selection of study areas where the community′s reliance on medicinal plants is most evident, ensuring both the presence of expert informants and the availability of the seeds being studied. Such criteria enhance the depth, reliability, and cultural validity of the data collected. The selection was further supported by recommendations from government officials, local stakeholders, and religious leaders during the preliminary survey period. During the preliminary survey, general information about the study area was gathered. Based on the information sampling technique, the sampled kebeles, the number of informants, and the study sites were determined.

### 2.5. Data Analysis

Data were analyzed using both descriptive and ethnobotanical quantitative methods. Data were checked for completeness, coded, and entered into EpiData Version 3.1 before being exported to SPSS Version 26.0 for statistical analysis. Descriptive statistics, including frequencies and percentages, were used to summarize the sociodemographic characteristics of the informants and patterns of medicinal use. Associations between categorical variables and patterns of medicinal seed use were assessed using the Pearson chi‐square test. Statistical significance was considered at *p* value of < 0.05. Ethnobotanical quantitative indices such as use value (UV), informant consensus factor (ICF), fidelity level (FL), and frequency of citation (FC) were used. The ICF was computed to identify potentially effective medicinal plant species in the respective disease categories [19]. Thus, ICF = (N_ur_ − N_t_)/(N_ur_ − 1), where ICF is the ICF, N_ur_ is the number of use reports in each category, and N_t_ is the number of species used. The preference of people for a certain species of medicinal plant for a given disease is shown in the FL [20]. The following equation was used to calculate it: FL (*%*) = Np/N × 100, where *N* is the total number of informants who cited plant species for various types of medicinal use, and *N* *p* is the number of informants who used plants as a medicine.

## 3. Results

### 3.1. Sociodemographic Characteristics of Respondents

A total of 65 individuals (23 males and 32 females) participated in the study. The majority of participants (32.3%) were aged between 30 and 39 years. By occupation, the majority of respondents (58.4%) were farmers and government employees, whereas merchants and other administrators together accounted for 41.6%. Most respondents (66.2%) were married, and 3.1% were divorced. Participants were grouped into four educational categories: illiterate, primary school completed, secondary school graduate, and college‐level education. The largest proportion (38.5%) had college‐level education, whereas 26.2% were illiterate (Table 1).

**Table 1:** Sociodemographic characteristics of respondents.

Variables	Category	Frequency (*N*)	Percentage (%)
Age	18–29	10	15.4
	30–39	21	32.3
	40–49	15	23.1
	≥ 50	19	29.2

Sex	Male	23	35.4
	Female	42	64.6

Marital status	Single	14	21.5
	Married	43	66.2
	Divorced	2	3.1
	Windowed	6	9.2

Educational level	Illiterate	17	26.2
	Primary	11	16.9
	Secondary	12	18.5
	University	25	38.8

Occupation	Farmer	19	29.2
	Merchant	16	24.6
	Government employee	19	29.2
	Nongovernment employee	1	1.5
	Other	10	15.4

Place of residence	Urban	45	69.2
	Rural	20	30.8

Roles	Traditional healer	2	3.1
	Herbal seller	28	43.1
	Experienced community member	18	27.7
	Other	17	26.2

Traditional healer work experience	11–15 years	1	1.5
	> 15 years	1	1.5

Abbreviations: *N*, number of respondents; percentage (%), proportion of respondents.

### 3.2. Treatment and Intervention Details

Regarding the medicinal use, majority of the respondents (93.8%) reported having used these seeds either for treatment or prevention purposes. Among those, 84.6% used both seeds in combination, whereas 9.2% and 6.2% used *N. sativa* and *L. sativum* individually, respectively. The primary source of information regarding these medicinal seeds was family members (64.6%), followed by information gained from the neighborhood (21.5%) and social media (10.8%), whereas traditional healers accounted for only 3.1%. Raw consumption was the most common method of seed preparation (55.4%), followed by powdered form (29.2%), whereas other methods such as mixing with food, boiling, or tea preparation were less frequent. The oral route (80.0%) was the dominant route of administration. Frequency of use varied, with 52.3% taking the remedies occasionally and 41.5% adhering to once‐daily. Regarding the duration of treatment, the majority of respondents (76.9%) typically consumed the remedies for less than 1 week (Table 2).

**Table 2:** Source of information, seed preparation and route of administration, and duration of treatment of *L. sativum* and *N. sativa* seeds.

Variables	Category	Frequency (*N*)	Percentage (%)
Have you used *L. sativum*/*N. sativa* seeds	Yes	61	93.8
	No	4	6.2

Type of seed used	*L. sativum*	4	6.2
	*N. sativa*	6	9.2
	Both	55	84.6

Source of information	Family member	42	64.6
	Traditional healer	2	3.1
	Neighborhood	14	21.5
	From social media	7	10.8

How long have you know their medical use	< 1 year	3	4.6
	5–10 years	8	12.3
	> 10 years	54	83.1

Seed preparation	Raw	36	55.4
	Powdered	19	29.2
	Boiled	4	6.2
	Tea	1	1.5
	Mixed with food	5	7.7

Root of administration	Oral	52	80.0
	Topical	2	3.1
	Other	11	16.9

How often do you take the remedies	Once daily	27	41.5
	Twice daily	2	3.1
	Three times daily	2	3.1
	Occasionally	34	52.3

For how long do you usually take	< 1 week	50	76.9
	1–4 weeks	5	7.7
	1–3 months	3	4.6
	> 3 months	7	10.8

Abbreviations: *L. sativum*, *Lepidium sativum*; *N*, number of respondents; *N. sativa*, *Nigella sativa*; percentage (%), proportion of respondents.

The pie chart represents majority of respondents (58.46%) use the whole raw seed, whereas nearly a third (30.77%) use the powder form. Oil extracts were significantly less common, accounting for only 9.23% of usage, with a negligible 1.54% use of other parts (Figure 1).

**Figure 1:**
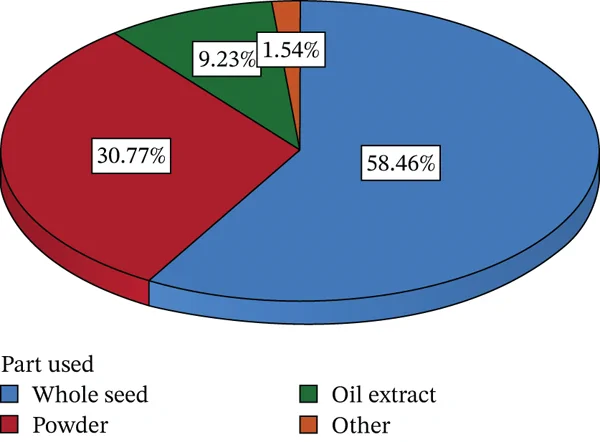
*Lepidium sativum* and *Nigella sativa* seeds used by respondents.

### 3.3. Dosage, Safety, and Perceived Effectiveness

The majority of participants prefer a combination of seeds over single‐seed treatments. The findings revealed that nearly half of the respondents consumed one teaspoon (46.2%), two teaspoons (16.9%), three teaspoons (15.4%), and four or more teaspoons (21.5%). Around 56.9% of the respondents determined the dose based on family advice, whereas 32.3% depended on personal experience. A smaller proportion gained guidance from traditional healers (7.7%), and very few (3.1%) reported having no fixed dose. The seeds were most commonly used for treating digestive problems (47.7%), followed by general health (38.5%). Fewer respondents reported using the seeds for hypertension (4.6%), respiratory illnesses (4.6%), pain (3.1%), and diabetes (1.5%).

The majority of respondents (78.5%) reported that the seeds are safe. However, some participants mentioned that pregnant women (24.6%), the elderly (10.8%), and individuals with chronic diseases (6.2%) should not use the seeds. The majority of respondents (83.1%) reported no side effects associated with seed use, whereas 16.9% indicated experiencing some side effects. Compared with modern medicine, 29.2% believed the seeds were more effective, 24.6% considered them to have similar effectiveness, and only 1.5% perceived them as less effective (Table 3).

**Table 3:** Dosage, safety, and perceived effectiveness of *L. sativum* and *N. sativa* seeds.

Variables	Category	Frequency (*N*)	Percentage (%)
Seed safety	Yes	51	78.5
	No	4	6.2
	Not sure	10	15.4

Who should not use	Pregnant women	16	24.6
	Elderly	7	10.8
	Chronic disease	4	6.2
	No restrictions	38	58.5

Compared with modern medicine	More effective	19	29.2
	Similar effectiveness	16	24.6
	Less effective	1	1.5
	Do not know	29	44.6

Prevention/treatment	Prevention	7	10.8
	Treatment	4	6.2
	Both	54	83.1

Dose determination	Family advice	37	56.9
	Traditional healer	5	7.7
	Personal experience	21	32.3
	No fixed dose	2	3.1

Amount usually takes	One teaspoon	30	46.2
	Two teaspoons	11	16.9
	Three teaspoons	10	15.4
	Four or more	14	21.5

Time until effects are observed	1 day	28	43.1
	A few days	31	47.7
	1 week	4	6.2
	After a week	2	3.1

Side effects	Yes	11	16.9
	No	54	83.1

Abbreviations: *N*, number of respondents; percentage (%), proportion of respondents.

### 3.4. Specific Disease Types and Related Plant Seeds Used in the Study Area

The bar graph illustrates the frequency of diseases reported to be treated by *L. sativum* and *N. sativa* seeds. Digestive problems were the most frequently mentioned, followed by general health. Lower frequencies were reported for diabetes, hypertension, respiratory illnesses, and pain/inflammation (Figure 2).

**Figure 2:**
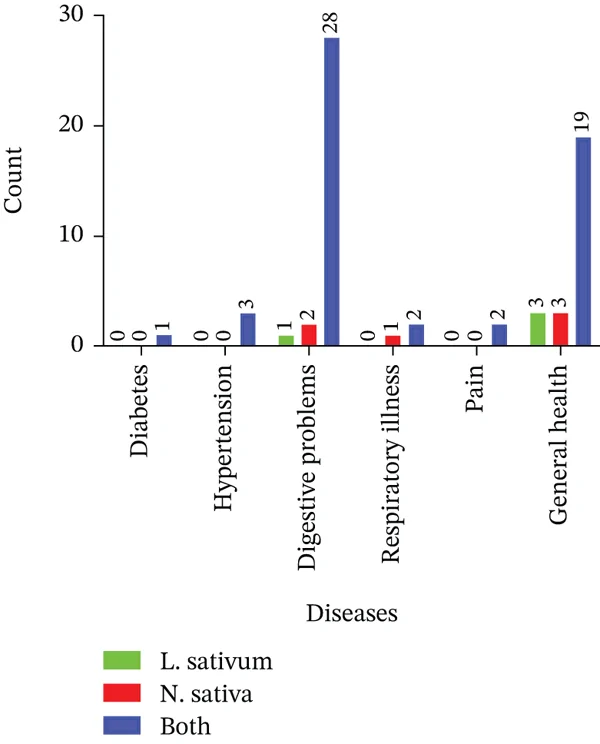
Disease treated by *Lepidium sativum* and *Nigella sativa* seeds.

The data presented in the bar graph represents that individuals use seeds in the morning either on a once‐daily or as‐needed basis. The morning time was the primary time for consumption, with 21 individuals mentioned for a once‐daily basis and 22 taking the seeds occasionally. Premeal consumption was the next most common time, favored by 10 users and 6 once‐daily users (Figure 3).

**Figure 3:**
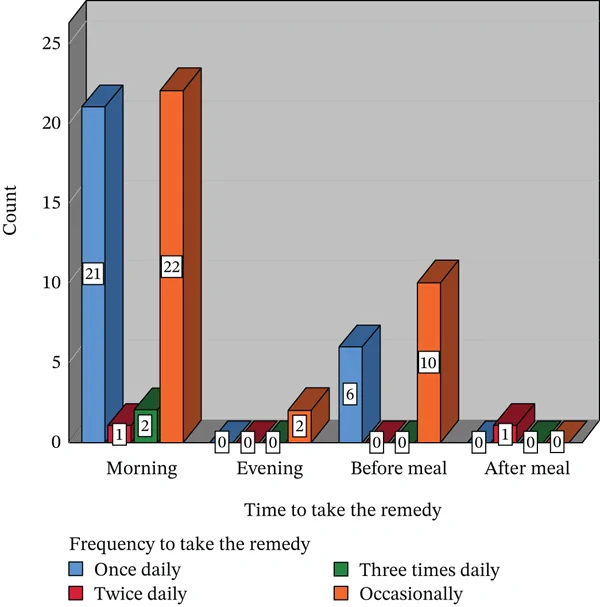
Timing and frequency of *Lepidium sativum* and *Nigella sativa* seeds consumption.

### 3.5. ICF per Disease Category

#### 3.5.1. Pearson Chi‐Square Test

A Pearson chi‐square test indicated a statistically significant association between pattern of medicinal seed use and age category, *χ* ^2^ (64), *p* = 0.045. This finding suggests that the pattern of medicinal seed use varied across age categories among the study participants (Table 4).

**Table 4:** Pearson′s chi‐square test.

Chi‐square tests			
	Value	df	Asymptotic significance (two‐sided)
Pearson chi‐square	84.411	64	0.045
Likelihood ratio	53.178	64	0.831
Linear‐by‐linear association	4.368	1	0.037
*N* of valid cases	65		

*Note:* *χ* ^2^ (64), *p* = 0.045, indicating a statistically significant association.

The ICF analysis showed that ICF values ranged from 0.000 to 0.933 across six disease categories. The highest consensus was recorded for digestive problems (ICF = 0.967) and general health (ICF = 0.958), with 31 and 25 use‐reports, respectively. These high values indicate a strong homogeneity in the ethnobotanical knowledge. In contrast, hypertension and respiratory illness showed moderate consensus, both yielding an ICF of 0.500. The lowest consensus was observed for pain/inflammation and diabetes, both of which resulted in an ICF value of 0.000 (Table 5).

**Table 5:** The informant consensus factor (ICF) values for the six disease categories.

Disease name	*N*_*u* *r*_	*N*_*t*_	*N*_*u* *r*_ − *N*_*t*_	*N*_*u* *r*_ − 1	ICF
Hypertension	3	2	1	2	0.500
Pain/inflammation	2	2	0	1	0.000
Digestive problems	31	2	29	30	0.967
General health	25	2	23	24	0.958
Respiratory illness	3	2	1	2	0.500
Diabetes	1	1	0	0	0.000

*Note:* ICF = (N_ur_–N_t_)/(N_ur_ − 1). Total informants (*N*) = 65.

Abbreviations: ICF, informant consensus factor; N_t_ number of species used; N_ur_, number of use reports in each category.

The effect of seed‐based treatments compared with modern medicine varies significantly across health conditions. Digestive problems and general health are the highest, with 10 and 5 respondents, respectively, rating the seeds as more effective than modern medicine. However, other respondents, (13) general health and (15) digestive problems, expressed uncertainty regarding the benefits. Similar effectiveness was reported by 6 individuals for digestive problems and 7 for general health, whereas other conditions, such as pain, respiratory illness, and hypertension, showed minimal comparative counts (Figure 4).

**Figure 4:**
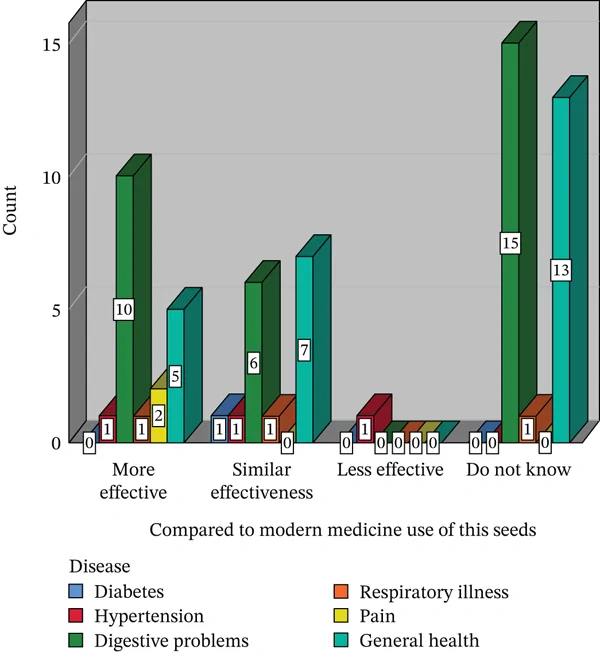
Perceived effectiveness of *Lepidium sativum* and *Nigella sativa* seeds compared with modern medicine.

The ethnobotanical indices showed a significant preference for the combined use of *L. sativum* and *N. sativa* with a FC value of 55 and a UV of 0.846. Digestive problems are found as the primary therapeutic indication, supported by 28 specific reports and a FL of 50.91%. *L. sativum* exhibited the highest degree of therapeutic specificity for general health, with a FL of 75.00%, despite a lower overall value of 0.062. In comparison, *N. sativa* showed a UV of 0.092, with a FL of 50.00% (Table 6).

**Table 6:** The frequency of citation (FC) and use value (UV) of respondents.

Plant seed type	FC	UV	Main disease treated	N_p_	FL %
Both (*L. sativum* and *N. sativa*)	55	0.846	Digestive problems	28	50.91%
*N. sativa*	6	0.092	General health	3	50.00%
*L. sativum*	4	0.062	General health	3	75.00%

Abbreviations: FC, frequency of citation; FL, fidelity level, FL = (Np/FC) × 100; *N*, total number of informants in the study (65); Np, the number of informants who specifically mention using a plant for a particular disease; UV, use value, UV = FC/N.

## 4. Discussion

In the study area, a total of six diseases were treated by *L. sativum* and *N. sativa*, where one species can treat a single disease or a number of diseases. Similarly, one disease can be treated with a combination of plant species or a single plant. The most commonly used mode of preparation was raw seed (55.4%,) followed by powdered (29.2%). In contrast to this study, other studies reported that the most commonly used mode of preparation was crushing, followed by pounding [21, 22].

Even though this study focused exclusively on the seeds of *L. sativum* and *N. sativa*, similar findings are also reported in other localities of Ethiopia, where seeds are the most widely used plant part for preparation [23]. In other localities of Ethiopia [24, 25], leaves are the most widely used plant parts for preparation. The dosage of the medicinal plants used in this study varies based on the diseases treated and among age groups and individuals. This finding is also similar to the study [26]. There is no standardized unit of measurement. This is a common problem in many areas of Ethiopia [27]. However, differences in the plants and preparation methods reported across regions may reflect variation in local flora, agroecological conditions, cultural transmission of medicinal knowledge, availability of plant materials, and differences in the health conditions prioritized by individual communities.

The ICF showed that ICF values ranged from 0.000 to 0.933 across six disease categories. The highest consensus was recorded for digestive problems (ICF = 0.933) and general health (ICF = 0.917), with 31 and 25 use‐reports, respectively. These high values indicate a great agreement among respondents. In contrast, hypertension and respiratory illness showed moderate consensus, both yielding an ICF of 0.500. The lowest consensus was observed for pain/inflammation and diabetes, both of which resulted in an ICF value of 0.000. These findings are similar to other studies that the ICF values, ranging from 0.47 to 0.92, with higher values indicating greater agreement among informants on plant use for specific conditions, such as the 0.92 ICF for gastrointestinal diseases and parasitic infections [28]. The ethnobotanical indices showed a significant preference for the combined use of *L. sativum* and *N. sativa* with a FC value of 55 and a UV of 0.846. Digestive problems are found as the primary therapeutic indication, supported by 28 specific reports and a FL of 50.91%. *L. sativum* exhibited the highest degree of therapeutic specificity for general health, with a FL of 75.00%, despite a lower overall UV of 0.062. Similar to this finding, another research paper revealed that *L. sativum* with an FL of 90% is cited for treating stomachache [29]. In contrast to this, another study revealed that *L. sativum* is the preferred remedy for treating febrile illnesses [30].

In comparison, *N. sativa* showed a UV of 0.092, with a FL of 50.00%. These results suggest that while both seeds are valued independently, their combination is the most significant practice for managing diseases.

## 5. Limitations

The primary limitation of this study was the small sample size, which may limit the generalizability of the findings to the wider community. Conducting large‐scale studies in future research would provide more comprehensive data.

## 6. Conclusion

This study demonstrates that *L. sativum* and *N. sativa* seeds remain important components of traditional health in the surveyed communities of North Shewa. The high level of reported use, particularly for digestive and general health, indicates that knowledge concerning these medicinal seeds remains actively maintained within the study communities. The observed variation in preparation methods, dosage, and indications also highlights the influence of local cultural practices and knowledge transmission. However, the reported traditional uses should not be interpreted as evidence of clinical efficacy or safety. Future studies should investigate the phytochemical composition, mechanisms of action, toxicological profiles, and clinical relevance of commonly used preparations of *L. sativum* and *N. sativa*.

## Author Contributions

D.M.M. was involved in the conception, design, analysis, interpretation, report, and manuscript writing. A.A. was involved in the conception, interpretation, report, and manuscript preparation. W.M. was also involved in the interpretation and report writing.

## Funding

No funding was received for this manuscript.

## Ethics Statement

Written ethical clearance was obtained from the research and ethical committee of the department of physiology Addis Ababa University. A formal letter was written to Debre Berhan Agricultural Research Institute and each kebele administration to conduct the study. Written informed consent was sought and obtained from every participant who decided to take part in the study. They were assured about the confidentiality of their responses.

## Consent

The authors have nothing to report.

## Conflicts of Interest

The authors declare no conflicts of interest.

## Data Availability

The data that support the findings of this study are openly available in [repository name] at [DOI].
